# Chemical and photonic interactions *in vitro* and *in vivo* between fluorescent tracer and nanoparticle-based scavenger for enhanced molecular imaging

**DOI:** 10.1016/j.mtbio.2019.100010

**Published:** 2019-06-12

**Authors:** T. Gulin-Sarfraz, E. Pryazhnikov, J. Zhang, L. Khiroug, J.M. Rosenholm

**Affiliations:** a Pharmaceutical Sciences Laboratory, Faculty of Science and Engineering, Åbo Akademi University, Turku, Finland; b Department of Pharmacy, University of Oslo, Oslo, Norway; c Neurotar LtD, Viikinkaari 4, 00790, Helsinki, Finland; d College of Bioengineering, Chongqing University, Chongqing 400044, China

**Keywords:** Silica nanoparticles, Imaging agents, Contrast enhancement, Tracer, Scavenger, Molecular imaging, Diagnostic imaging

## Abstract

We hereby present a concept of scavenging excess imaging agent prior to a diagnostic imaging session, consequently allowing for enhanced contrast of signals originating from the tissue area of interest to the signals originating from systemic imaging agent residues. In our study, a prospective silica core–shell nanoparticle-based scavenger was designed and explored for its feasibility to scavenge a specific imaging agent (tracer) in the bloodstream. The developed tracer–scavenger system was first investigated under *in vitro* conditions to ensure proper binding between tracer and scavenger is taking place, as confirmed by Förster/fluorescence resonance energy transfer studies. *In vivo*, two-photon imaging was utilized to directly study the interaction of the scavenger particles and the tracer molecules in the vasculature of mice. To our knowledge, a methodological solution for *in vivo* differentiation between signals, originating from tissue and blood, has not been presented elsewhere.

## 1 Introduction

Non-invasive biomedical and medical imaging techniques are powerful tools for diagnosis of various diseases, as well as monitoring disease progression and response to therapy. The rapidly evolving field of molecular imaging [1], [2], [3], [4], [5], [6] requires administration of a contrast agent prior to an imaging procedure in order to improve the quality of the generated images [7]. Contrast agents utilized for imaging purposes are substances that temporarily change the way electromagnetic radiation interact with internal structures or tissues [8], thus making them appear different on the images. While certain contrast agents are capable of crossing intact blood–tissue barriers, leakage of the contrast agent from blood to surrounding tissue is significantly more pronounced when a blood–tissue barrier is disrupted. Disruption of blood–tissue barriers occurs in a variety of tumors and diseases of the central nervous system and of the cardiovascular system and also accompanies inflammation and physical injuries [9], [10]. Hence, leakage of a contrast agent from blood to tissue at a particular site and accumulation thereto may be indicative of any of these pathological conditions. However, a major drawback associated with conventional molecular imaging methods and contrast agents utilized therewith is failure to create sufficiently high contrast between vasculature and surrounding tissue. Thus, conventional imaging methods often fail to accurately differentiate between the emitted signals originating from the contrast agent circulating in the blood flow and the emitted signals originating from the contrast agent retained in the tissue. As a result, efficient detection of structural changes indicative of a disease is limited to tissues where such changes have already affected relatively large regions. Certain pathological conditions are therefore medically diagnosed at a rather late stage [11], [12], [13], [14]. Consequently, there is an increasing need for a novel method and/or an appliance allowing for *in vivo* differentiation between signals originating from the blood circulation and signals originating from surrounding tissue. This would allow for developing a medical imaging technology that provides a radically improved contrast between tissue and blood. As a result, most of the neurodegenerative, inflammatory, and cancer-related disorders could be detected and characterized at earlier stages.

**Fig. 1:**
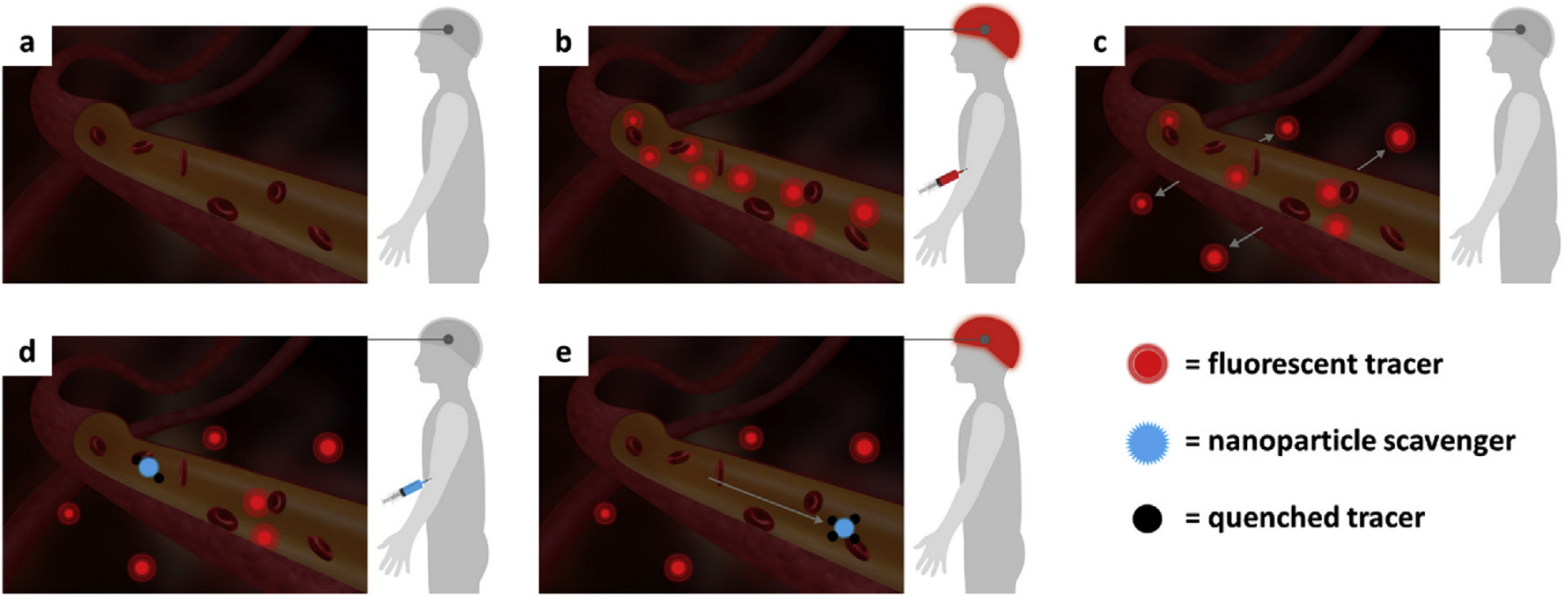
**Schematic illustration of the tracer–scavenger concept.** (a) Before the imaging session; (b) tracer is administered intravenously (‘time zero’), and baseline image is collected; (c) tracer molecules are partially redistributed through vessel walls to the brain tissue; (d) nanoparticles are administered to scavenge (bind) and quench the tracer, and (e) once all background fluorescence in blood vessels is eliminated, imaging is resumed.

To address this issue, we have developed a tracer–scavenger system to investigate the ability of using silica nanoparticles (NPs) to scavenge a specific tracer compound and quench its fluorescence directly in the blood circulation. The concept is schematically presented in Fig. 1, where brain imaging has been used as example. Here, molecular imaging agents can be designed to cross the blood-brain barrier (BBB), whereas larger NP constructs in general would not permeate the BBB. The scavenging strategy is therefore, in essence, two-fold: along with the quenching of fluorescence upon scavenging, drastic alteration of the biodistribution of the tracer compound would be expected after NP binding, which should further aid the imaging process. For the conceptual presentation of such a scavenger system, we selected the high-affinity biotin–streptavidin (STV) binding system as the molecular linker, because this is one of the strongest interactions known in nature [15]. This binding pair has also in practice been successfully used in many applications, including microarray technology [16] and development of bioassays [17] and biosensors [18]. Similarly, silica NPs have, to date, been intensively investigated for a range of different biomedical applications, including diagnostic and therapeutic interventions [19], [20], [21], [22], [23]. Notably, non-porous fluorescent silica NPs ‘C-dots’ were approved for clinical trials in 2010 for cancer diagnostics [24], and the design aspects of silica materials specifically for imaging are considerably vast [25], [26], [27]. The applicability, robustness, functionality, biocompatibility, and bioerosion rate as well as other crucial properties of silica particles in a biological environment can be tuned by a variety of surface functionalizations, which simultaneously provide reactive sites for further attachment of bioactive molecules. Additionally, silica shells can be utilized to protect other types of core materials sensitive to the biological milieu [28], [29], [30], while porous silica shells can be utilized for accommodating cargo molecules and/or maximizing the surface area available for further surface functionalization. For this study, we used our previously developed non-porous@porous core@shell silica particles as a model because these have the optimal size (approximately 200 nm) to serve the purpose of, on one hand, not be able to bypass the BBB even if disrupted and, on the other hand, stay in the circulation for enough time to exert their scavenger function. The particles have a solid silica core, representative of any inorganic colloidal material, which could be changed depending on the further application prospects, e.g., to a magnetic core [31] for a magnetic tracer removal system that could itself be traced with magnetic resonance imaging (MRI). Here, the mesoporous silica shell serves as a functionalization platform for growing a high density of reactive amino groups for further attachment of STV on the particle surface. Biotin-labeled dextran was used as model tracer compound. The STV and the dextran were both fluorescently labeled as a Förster/fluorescence resonance energy transfer (FRET) pair, with Dylight 549 and fluorescein in the form of fluorescein isothiocyanate (FITC), respectively. As a result, the observed FRET reports on the interaction between the scavenger particles and the tracer compounds, as demonstrated *in vitro*. A real-time physicochemical interaction between the tracer and scavenger was further investigated directly in the bloodstream of mice, where a statistically significant shift of fluorescence ratio towards the scavenger was observed *in vivo*.

## 2 Experimental methods

### 2.1 Preparation of silica core–shell particles, *n*SiO_2_@*m*SiO_2_

The silica core@shell particles, *n*SiO_2_@*m*SiO_2_, were synthesized according to our earlier protocol as reported in the study by Gulin-Sarfraz et al. [32]. Briefly, 100-mL ethanol (99%), 15-mL Milli-Q water, and 1.45-mL ammonium hydroxide (NH_4_OH, 33 wt%) were mixed, after which 7-mL tetraethyl orthosilicate (TEOS, ≥98%) was added and the solution was stirred for 24 h. The obtained silica cores were separated by centrifugation, washed with ethanol and acetone, and dried in a vacuum oven. 0.1-g Pluronic P123 (triblock-co-polymer, EO_20_PO_70_EO_20_) and 1.16-g sodium chloride (NaCl, 99.7%) were dissolved in 80-mL Milli-Q water and 32-mL ethanol. 0.3-g silica cores were added, and the solution was stirred at 35 °C for 30 min. Further 0.45-mL TEOS was added, and the stirring was continued for 24 h. The nSiO_2_@mSiO_2_ particles were separated by centrifugation, and the P123 template was extracted by sonication with acetone 3 times for 30 min.

### 2.2 PEI functionalization and succinylation of the particles, *n*SiO_2_@*m*SiO_2_@PEI and *n*SiO_2_@*m*SiO_2_@PEI@succ

For poly(ethylene imine) (PEI) functionalization, 0.3-g *n*SiO_2_@*m*SiO_2_ particles were vacuum-dried at 45 °C for 4 h after which they were dispersed in toluene (99.8%) under inert atmosphere. 150 μL aziridine (98%) and 15 μL acetic acid (CH_3_COOH, ≥99.8%) were added, and the reaction mixture was stirred at 75 °C for 24 h. The particles, *n*SiO_2_@*m*SiO_2_@PEI, were separated by centrifugation at 7500 rpm 10 min, washed by acetone, and vacuum-dried.

For succinylation, 0.05 g *n*SiO_2_@*m*SiO_2_@PEI particles were dispersed in dimethylformamide (≥99.9%). 0.1 g succinic anhydride was added, and the mixture was stirred for 24 h. The *n*SiO_2_@*m*SiO_2_@PEI@succ particles were separated by centrifugation at 10000 rpm 10 min, washed by acetone and ethanol, and finally vacuum-dried.

### 2.3 STV conjugation to the particles, *n*SiO_2_@*m*SiO_2_@PEI@STV and *n*SiO_2_@*m*SiO_2_@PEI@succ@STV

10 mg *n*SiO_2_@*m*SiO_2_@PEI or *n*SiO_2_@*m*SiO_2_@PEI@succ particles were dispersed in 2-(N-morpholino)ethanesulfonic acid (MES) buffer (100 mM, pH 5). 0.25 mg DyLight-549-STV was also suspended in MES buffer, to which 10 μL 1-ethyl-3-(3-dimethylaminopropyl) carbodiimide (EDC) was added to activate the carboxylic acid groups of STV. Further 0.2 mg N-hydroxysuccinimide (NHS) was added, and the whole mixture was added to the particle suspension. The suspension was left to react under stirring at 6 °C for 7 h. The particles were separated by centrifugation at 2500 rpm 10 min, washed by 4-(2-hydroxyethyl)-1-piperazineethanesulfonic acid (HEPES) buffer (25 mM, pH 7.2) and finally dispersed in HEPES.

### 2.4 Particle characterization

Transmission electron microscopy (TEM) measurement was carried out with a JEOL-2200FS microscope, operated at 200 kV. Dynamic light scattering and zeta potential measurements were performed on a Malvern Zetasizer Nano ZS instrument, by dispersing the particles in HEPES. The amount STV adsorbed to the particles was determined with UV absorbance at 280 nm wavelength by stepwise addition of STV to the particle-suspension in HEPES, while the supernatant was measured on a Nanodrop2000c UV-VIS spectrophotometer. The photonic interaction (in terms of FRET) between the DyLight-549-STV-conjugated particles and the fluorescein-biotin-dextran (10.000 MW) tracer was determined by stepwise addition of STV-particles to dextran suspended in HEPES, and the fluorescence (λ_ex_ = 488 nm, λ_em_ = 500–600 nm) was recorded on a PerkinElmer LS50B luminescence spectrometer.

### 2.5 Two-photon *in vivo* imaging

All animal procedures were performed in accordance with the University of Helsinki animal care regulations. Local authority (ELÄINKOELAUTAKUNTA-ELLA) approved the animal license (ESAVI/9071/04.10.07/2016) to conduct the procedures described in the study.

2 female C57BL/6JRccHsd WT mice were used for the imaging. 3–4 weeks before the start of imaging experiments, animals were anesthetized with ketamine/xylazine and operated for implantation of a cranial window. The cranial window was inserted over the somatosensory cortex at the following coordinates: AP −1.8, ML −2.0 from Bregma. Dental drill was used to remove a round shaped (d = 4 mm) piece of skull, and the hole in the bone was covered with a round cover glass (d = 5 mm). Mice were imaged with the FV1200MPE two-photon microscope (Olympus, Japan) with the 25X water immersion 1.05 NA objective specially designed for *in vivo* two-photon imaging. MaiTai Broad Band DeepSee laser tuned to 800 nm was used for excitation. In a preliminary set of *in vitro* tests 800 nm was found to be optimal for preferential excitation of FITC fluorophore. Unfortunately, isolated excitation of the two fluorophores was not possible due to the limitations of two-photon excitation and lasers. Emission light was collected using the band pass filters: 515–560 nm for FITC fluorescence and 590–650 for Dylight 549 fluorescence.

For *in vivo* imaging sessions, animals were anesthetized with ketamine/xylazine and head-fixed under two-photon microscope. Three-dimensional (3D) baseline autofluorescence image Z-stacks of the mouse somatosensory cortex was acquired. Stacks of images were collected with the vertical step of 3 μm with zoom factor one at 800 × 800 pixels aspect ratio. Line-scans were performed inside the lumen of superficial cortical vessels before and after the injection of biotin-labeled (or unlabeled) dextran and STV-labeled NPs (*n*SiO_2_@*m*SiO_2_@PEI@STV). 100 μL of 10 mg/mL solution of conjugated NPs was injected intravenously.

After collection of the data, images were analyzed using Fiji/ImageJ software. NPs were visually detected in the Dylight 549 channel and analyzed by ratiometric analysis. The advantage of using ratiometric analysis is that it is not sensitive to crosstalk between the dyes as crosstalk is constant between the experiments. Background fluorescence was subtracted from all the images. In total 19 NPs were analyzed. Graphs were plotted in Microcal Origin software.

## 3 Results and discussion

### 3.1 Development of the scavenger NPs

**Fig. 2:**
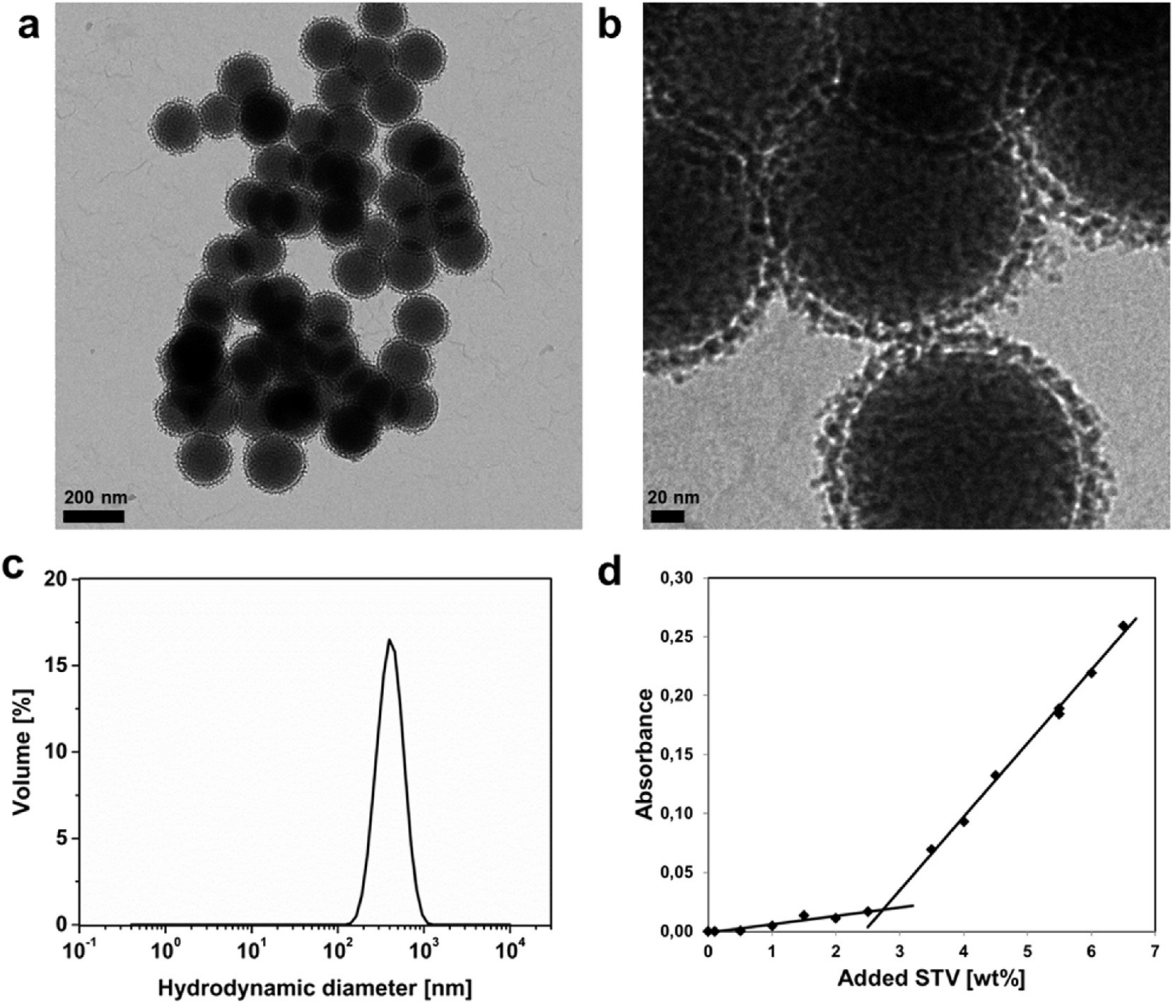
**Characterization of the produced nanoparticles.** (a & b) Transmission electron microscopy images of the *n*SiO2@*m*SiO2 particles show monodisperse particles coated with a thin porous shell. (c) Dynamic light scattering reveals well-dispersed particles in buffer solution (HEPES, pH 7.2) with a hydrodynamic diameter of 345 nm and PdI of 0.09, as analyzed from three consecutive measurements. (d) UV absorbance measurement for determining protein concentration in the supernatant after streptavidin (STV) addition to the particle suspension (λ = 280 nm), shows a strong (complete) adsorption of STV to the particles up to 2.5 wt%, whereafter unspecific adsorption of STV with less affinity to the particle surface most likely takes place. HEPES, 4-(2-hydroxyethyl)-1-piperazineethanesulfonic acid.

As prospective particle platform for the scavenger system, our previously developed [32] non-porous@porous core@shell silica particles (*n*SiO_2_@*m*SiO_2_) were chosen. These particles have a solid silica core and a thin mesoporous silica shell, which increases the available surface area for growing a high density of reactive amino groups. The surface area of the particles was approximately 100 m^2^/g, as measured by nitrogen sorption and presented in Supplementary Fig. S1. The solid core is representative of any inorganic colloidal material, and may thus easily be exchanged to i.e. a magnetic core for magnetic manipulation or any other inherently detectable core for imaging. In case of a magnetic core, the size of the core is optimal for both a strong magnetization and a high contrast in MRI, as previously demonstrated [31]. The size of particles for a specific application has to be carefully chosen, since this, along with surface functionalization, is crucial for the biodistribution of the particles [33]. It is generally known that particles with a diameter less than 200 nm exhibit prolonged blood circulation time and are more favorable for crossing disrupted blood–tissue barriers (‘passive targeting’) via the enhanced permeation and retention (EPR) effect [34]. Thus, for this study we chose the size of the particles for optimum circulation time, but still large enough to rather stay in the blood circulation than easily extravasate into the leaky vasculature through disrupted blood–tissue barriers. TEM images (Fig. 2 a and b) revealed monodisperse core–shell particles, with a size of 150–200 nm. The size distribution analysis from TEM is presented in Supplementary Fig. S2. These negatively charged particles (zeta potential −32 mV) were amino-functionalized with a surface-grafted hyperbranched PEI layer, *n*SiO_2_@*m*SiO_2_@PEI, whereby they obtained a high positive charge (+41 mV) in HEPES buffer (25 mM, pH 7.2). The surface-functionalized particles were fully dispersible in HEPES buffer with a hydrodynamic diameter peak centered on 350 nm (Fig. 2c).

For our prospective scavenger system, STV was attached onto the particle surface to specifically recognize and bind the biotin-labeled dextran used as tracer. Since STV is negatively charged at physiological pH and has many functional groups, including carboxylic acids (-COOH), it can easily be either adsorbed onto the positively charged *n*SiO_2_@*m*SiO_2_@PEI particle surface or covalently linked to the primary amines (-NH_2_) via amide bonds. To obtain some insight on the amount of STV that can be attached to the particle surface, STV was adsorbed onto the particles by stepwise addition (titration) whereafter the protein concentration left in the supernatant was measured by UV absorbance. Fig. 2d shows a complete adsorption of STV to the particles up to approximately 2.5 wt% with respect to the particle weight. This amount of STV (2.5 wt%) was consequently used for covalently labeling the particles via EDC-NHS coupling, resulting in *n*SiO_2_@*m*SiO_2_@PEI@STV particles. The decrease in zeta potential from +41 mV for the *n*SiO_2_@*m*SiO_2_@PEI particles to +35 mV for the *n*SiO_2_@*m*SiO_2_@PEI@STV particles further indicated that the negatively charged STV had been conjugated to the surface (Supplementary Fig. S3).

**Fig. 3:**
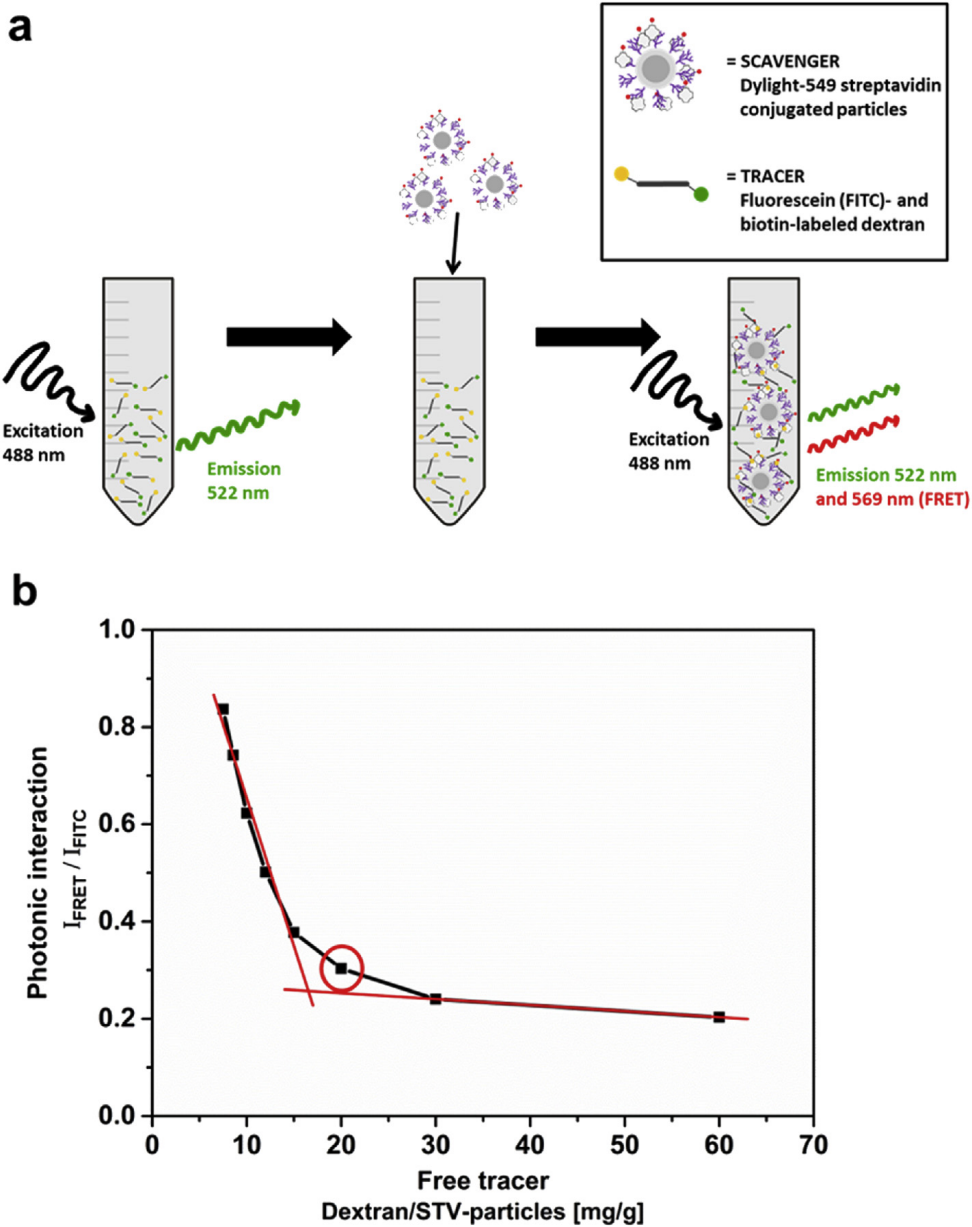
**Interaction between dextran tracer and scavenger particles by streptavidin–biotin binding as measured by FRET.** (a) The initial solution of fluorescein- and biotin-labeled dextran gives rise to green emission only, while after addition of Dylight 549-STV conjugated particles, the green emission decreases and yellow emission increases. (b) The photonic interaction, in terms of FRET (I_FRET_/I_FITC_), increases with higher amount of particle-bound tracer. The lines are added as visual guides to indicate the correlation. The circled point corresponds to a molar ratio of 2:1 of dextran and streptavidin.

For the tracer–scavenger system, biotin-labeled dextran (10.000 MW) was chosen as model tracer compound. The system was first investigated under *in vitro* conditions to ensure binding between tracer and scavenger is taking place, as confirmed by FRET studies (schematically described in Fig. 3a). STV and dextran were thus both fluorescently labeled as a FRET-pair, with fluorescein-labeled dextran acting as FRET-donor and Dylight 549-labeled STV as acceptor. The initial solution of fluorescein- and biotin-labeled dextran only gave rise to green emission (520 nm), while after stepwise addition of Dylight 549-STV conjugated particles (*n*SiO_2_@*m*SiO_2_@PEI@STV) the green emission decreased and yellow emission (570 nm) increased (Fig. S2a†). The results are presented in Fig. 3b and show a clear correlation between the photonic interaction in terms of FRET (*I*_FRET_/*I*_FITC_), and the amount of free dextran tracer, where the photonic interaction readily increases when the amount of free tracer approaches zero. The circled point, after which the interaction becomes pronounced as evidenced by the steep rise in the slope and a breaking point between concentration ratio points 15 and 20 mg/g, correlates to a molar ratio of 2:1 of dextran and STV, respectively (Fig. 3b and Supplementary Fig. S4b). STV is a rectangular-shaped tetrameric protein with four biotin binding sites [35]. Given that the STV in this case is immobilized onto a particle, it would be expected that not all four of the biotin binding sites will be accessible for binding with the dextran-conjugated biotin molecules.

Here, we note that the choice of FRET pair was based on their well-established interaction that could be illustratively used to demonstrate our concept. However, for real-life applications, this tracer–scavenger pair may be less ideal. Fluorescence-based quantifications are a challenge as such and further associated with complications related to both the surroundings (e.g. pH, polarizability) and the most immediate environment. In the latter case the particles themselves are regarded, since the scavenger dyes are in close proximity of the particle surface. For instance, amino groups typically favor energy (charge) transfer mechanisms whereas amide bond formation would generally be used for conjugation reactions. Thus, amine groups may most likely be present on a scavenger particle surface, which may lead to proton energy transfer yielding efficient emission in the blue/green spectral region. Based on the mode of incorporation, particle immobilization of dyes may shift the emission peak, quench neighboring dye molecules, alter the pH of the immediate surrounding of the dye and so forth [23], [27], [36]. In the present case, the scavenger dyes are conjugated to the STV and not directly to the particles, and thus, the particle-associated effects should be of less importance. Nevertheless, our interpretations are still made on a qualitative level only due to the limitations associated with fluorescence intensity measurements, which should be taken into account upon further optimization of the tracer–scavenger system.

It has been shown that functionalizing the PEI coating with succinic acid groups (‘succinylation’) decreases protein adsorption on the particles [36], since the zwitterionic nature of this coating provides better stealth properties and the particles might hence circulate in the blood for a longer period of time. Therefore, the *n*SiO_2_@*m*SiO_2_@PEI particles were further succinylated, and the subsequent *n*SiO_2_@*m*SiO_2_@PEI@Succ particles acquired a zeta potential of −48 mV. Upon addition to the particle suspension, no STV was adsorbed onto the negatively charged *n*SiO_2_@*m*SiO_2_@PEI@Succ particles, most likely due to electrostatic repulsion between the STV and the particles (Supplementary Fig. S5). Even if STV did not physically adsorb on the particles, they could still be covalently conjugated with STV (*n*SiO_2_@*m*SiO_2_@PEI@Succ@STV), although not to the same degree as the *n*SiO_2_@*m*SiO_2_@PEI particles (Supplementary Fig. S6a). Consequently, the *n*SiO_2_@*m*SiO_2_@PEI@Succ@STV particles were deemed as a less efficient scavenger system for the dextran tracer than the non-succinylated *n*SiO_2_@*m*SiO_2_@PEI@STV particles in this case (Supplementary Figs. S6b and S6c), and therefore the *n*SiO_2_@*m*SiO_2_@PEI@STV particles where chosen for further *in vivo* studies.

### 3.2 Chemical and photonic interaction imaged *in vivo* in the blood circulation of mice

**Fig. 4:**
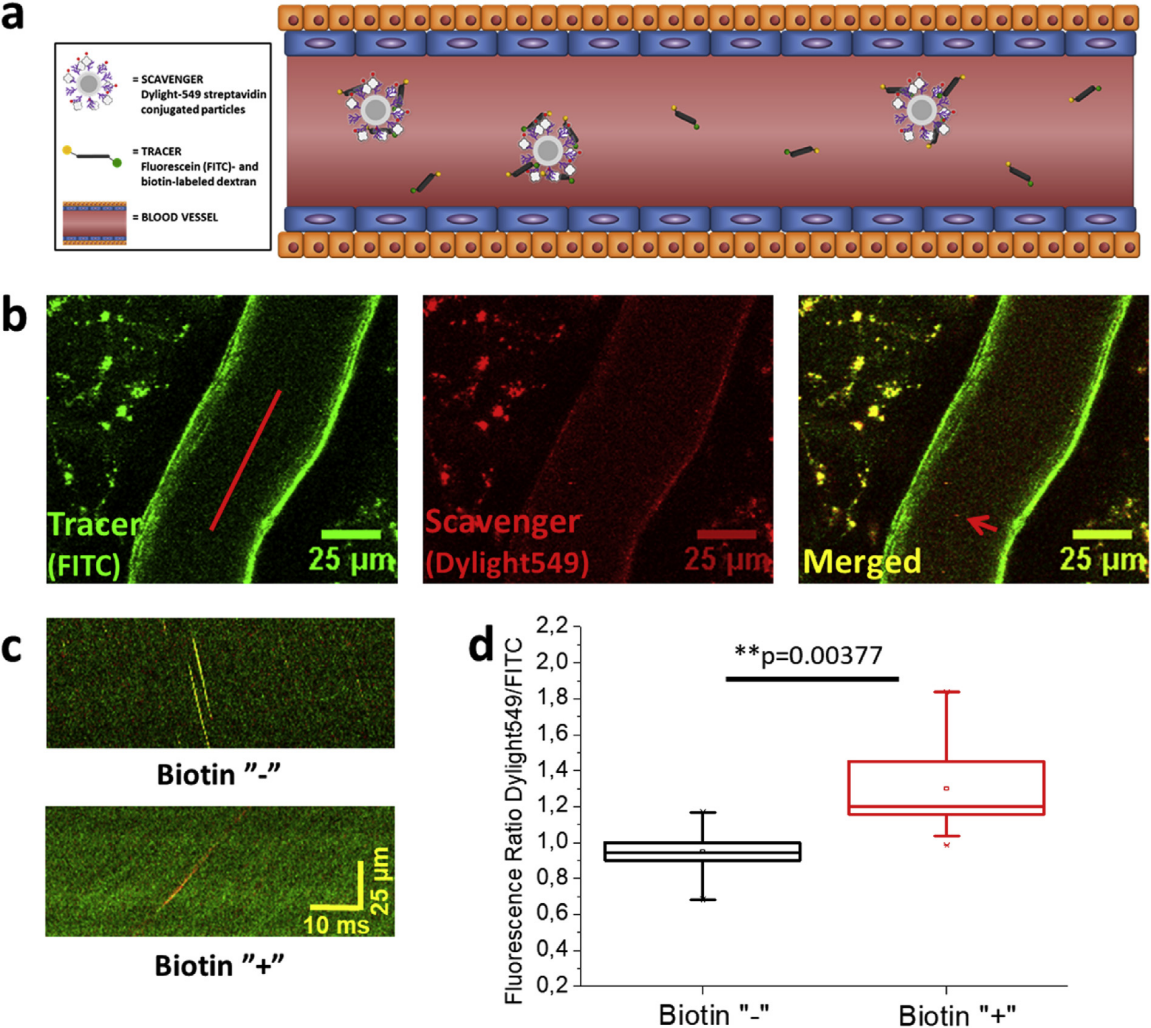
**Interaction between dextran tracer and silica scavenger particles by streptavidin–biotin binding *in vivo*.** (a) Schematic illustration of the interaction between tracer and scavenger in a blood vessel. (b) Examples of mouse cortical vessels used for visualization of tracer; fluorescein (FITC)- and biotin-labeled dextran (left panel), and scavenger; Dylight549- and streptavidin-labeled nanoparticles (middle panel) *in vivo*. Merged image is shown in the right panel. Red line in the left panel represents a line-scan along the vascular lumen used to detect and analyze nanoparticles. Red arrow in the right panel shows example of nanoparticle detected in the typical 2D plane image. (c) Examples of nanoparticles detected with line profile scanning, where the tracer (FITC) and scavenger (Dylight549) channels are superimposed. The images comprise stacks of individual line-scans (as shown in (b)), with time shift on the x-axis and distance on y-axis, resulting in nanoparticles detected in the form of lines. The lower image represents an experiment where the dextran tracer is labeled with biotin (Biotin ‘+’), while the upper image presents a control experiment where the tracer has no biotin (Biotin ‘-’). A more reddish hue of nanoparticles in the presence of biotin is noted. (d) Analysis of the change in the ratio between FITC and Dylight549 channels in experiments with (Biotin ‘+’) and without (Biotin ‘-’) biotin. In the presence of biotin on the dextran tracer we observed statistically significant shift of ratio towards scavenger (STV-Dylight549) channel highlighting physical and chemical interaction between dextran tracer and silica scavenger particles *in vivo*.

*In vivo*, two-photon imaging was used to directly study the chemical and photonic interactions of the scavenger particles and the tracer molecules in the bloodstream of mice (as schematically illustrated in Fig. 4a). To visualize the interaction of Dylight 549-STV conjugated particles (*n*SiO_2_@*m*SiO_2_@PEI@STV) and fluorescein- and biotin-labeled dextran, we implanted chronic cranial windows over mouse somatosensory cortex. Shortly after intravenous injection of Dylight 549-STV conjugated particles, we were able to visualize them in the mouse cortical vessels (Fig. 4b). As NPs moved in the blood circulation with a high speed, the best approach to detect and analyze them was to use line-scans with high acquisition rate to catch and trace them (Fig. 4c).

Since the dextran tracer filled most of the available volume of blood vessels, and thus the tracer fluorescence (fluorescein) was more pronounced, the best approach for detection of the chemical and photonic interaction between tracer and scavenger was to observe the increase in the ratio of fluorescence between the Dylight549 and fluorescein channels on individual NPs. This is analog to the increase in acceptor fluorescence observed in the FRET photonic interaction when conditions for photonic interaction are optimal. Indeed, we found a statistically significant shift in fluorescence towards the scavenger fluorescence (Dylight549 channel) in the experiment with biotin-fluorescein–labeled tracer *vs* experiment where the tracer was only labeled with fluorescein and no biotin (*p* = 0.00377), as shown in Fig. 4d. This means that the strong interaction between biotin and STV led to a detectable physicochemical interaction between the tracer and scavenger *in vivo*.

## 4 Conclusion

This conceptual study highlights the possibilities for a novel method of removing excess contrast media from the blood circulation prior to a diagnostic imaging session, to improve the contrast between tissue and blood. Here, silica core@shell NPs were used as scavenger platform, and the strong STV–biotin binding pair acted as model system for any other high-affinity ligand interaction. The STV-functionalized particles could, consequently, recognize and bind the biotin-labeled dextran tracers *in vitro* and *in vivo*. The particle-scavenger and dextran-tracer were labeled as a FRET pair, whereby the binding within the scavenger–tracer system could be demonstrated by FRET under *in vitro* conditions. After intravenous injection of the tracer compound in mice, with subsequent injection of the scavenger, the interaction between these could be visualized in the cortical vessels by two-photon microscopy. This demonstration shows the capability of the NPs to scavenge specific tracer compounds directly in the blood circulation, which can provide for an efficient method to remove and/or quench excess contrast agents prior to an imaging session. This would allow for better contrast of signals originating from the tissue area of interest and the residual signals originating from the blood circulation. Ultimately, this approach could increase the probability of earlier and more accurate diagnosis of various diseases.

## Declaration of interests

The authors declare that they have no known competing financial interests or personal relationships that could have appeared to influence the work reported in this paper.
