# Supplementary material for: Chemical and photonic interactions in vitro and in vivo between fluorescent tracer and nanoparticle-based scavenger for enhanced molecular imaging
Source: Mater Today Bio. 2019 Jun 12;2:100010. doi: 10.1016/j.mtbio.2019.100010 (PMC7061632; doi:10.1016/j.mtbio.2019.100010)
Supplement: Supplementary file 1 [file mmc1.docx]

**Supplementary information:**

**Chemical and Photonic Interactions *in vitro* and *in vivo* between Fluorescent Tracer and Nanoparticle-based Scavenger for enhanced Molecular Imaging**

Tina Gulin-Sarfraz^1,2^, Evgeny Pryazhnikov^3^, Jixi Zhang^4^, Leonard Khiroug^3^* and Jessica M. Rosenholm^1^*

1. Pharmaceutical Sciences Laboratory, Faculty of Science and Engineering, Åbo Akademi University, Turku, Finland
2. Department of Pharmacy, University of Oslo, Oslo, Norway
3. Neurotar LtD, Viikinkaari 4, 00790, Helsinki, Finland
4. College of Bioengineering, Chongqing University, Chongqing 400044, China

**Additional figures:**

**
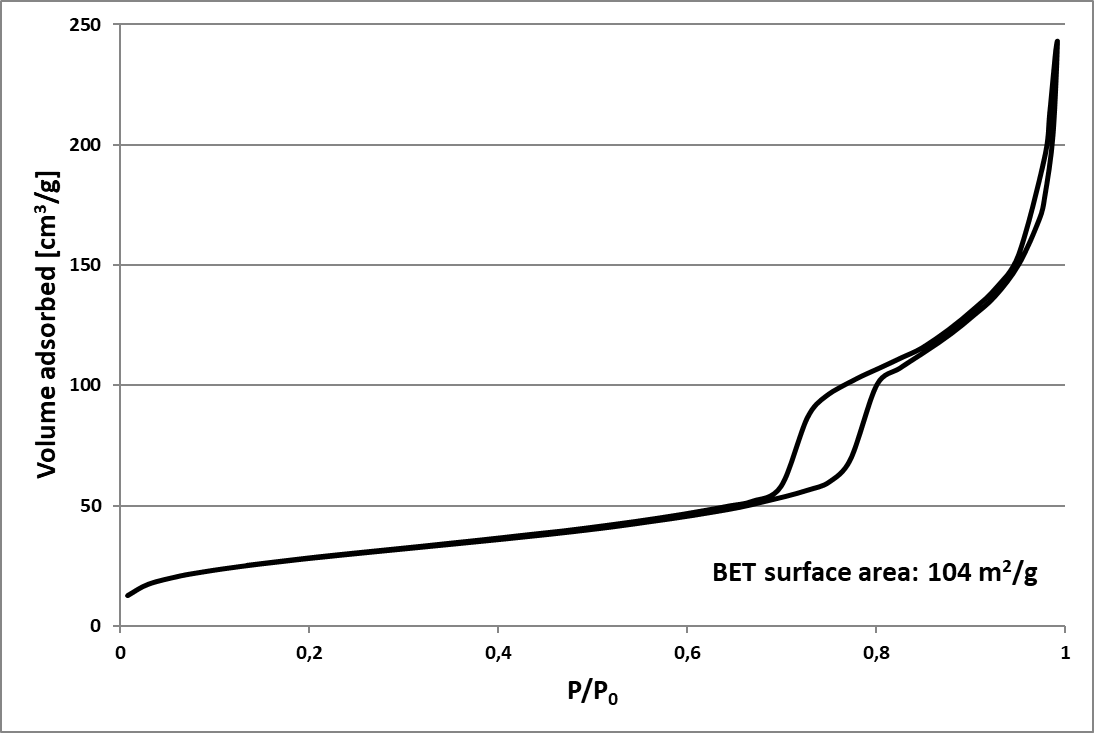
**

**Fig. S1** Nitrogen sorption isotherm of *n*SiO_2_@*m*SiO_2_ particles.

**
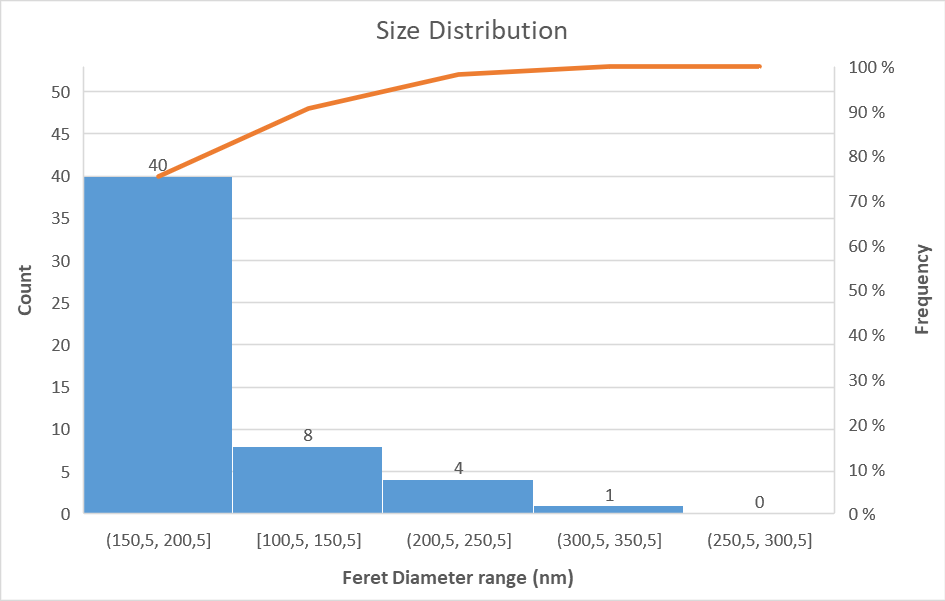
**

**Fig. S2** Particle size distribution analysis of *n*SiO_2_@*m*SiO_2_ particles from TEM images. The Feret diameter was analyzed for 53 nanoparticles by ImageJ.

**Fig. S3** Electrokinetic measurements (zeta potential) of the untreated nSiO_2_@mSiO_2_@PEI particles and the streptavidin-labeled particles, nSiO_2_@mSiO_2_@PEI@STV. The zeta potential of the untreated particles, +41 mV, dropped to +35 mV when labeled with negatively charged streptavidin.


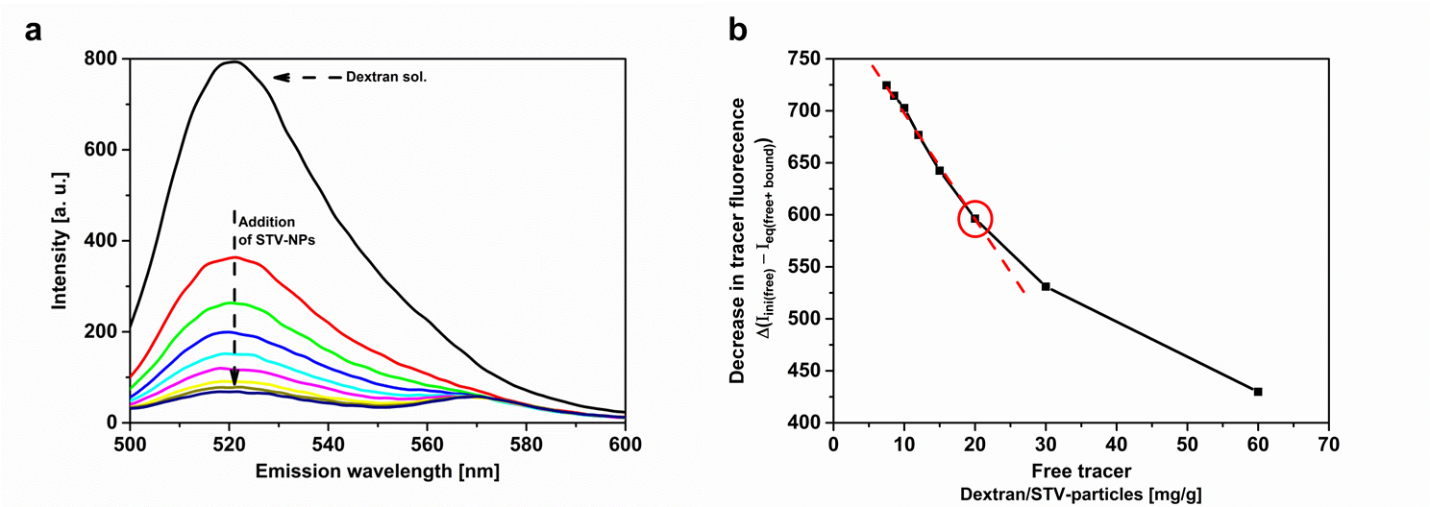


**Fig. S4** (a) Fluorescence intensity measurements of the initial dextran (tracer) solution, and after each addition of streptavidin-labeled (scavenger) particles. Emission of FITC at 520 nm decreases with each addition, while the FRET emission intensity at 570 nm increases (a, raw data to Fig. 3b and Fig. S3b). (b) With addition of streptavidin-labeled scavenger particles, the amount of free dextran decreases resulting in decreasing tracer fluorescence. The circled point correlates to a molar ratio of 2:1 of dextran and streptavidin, respectively.


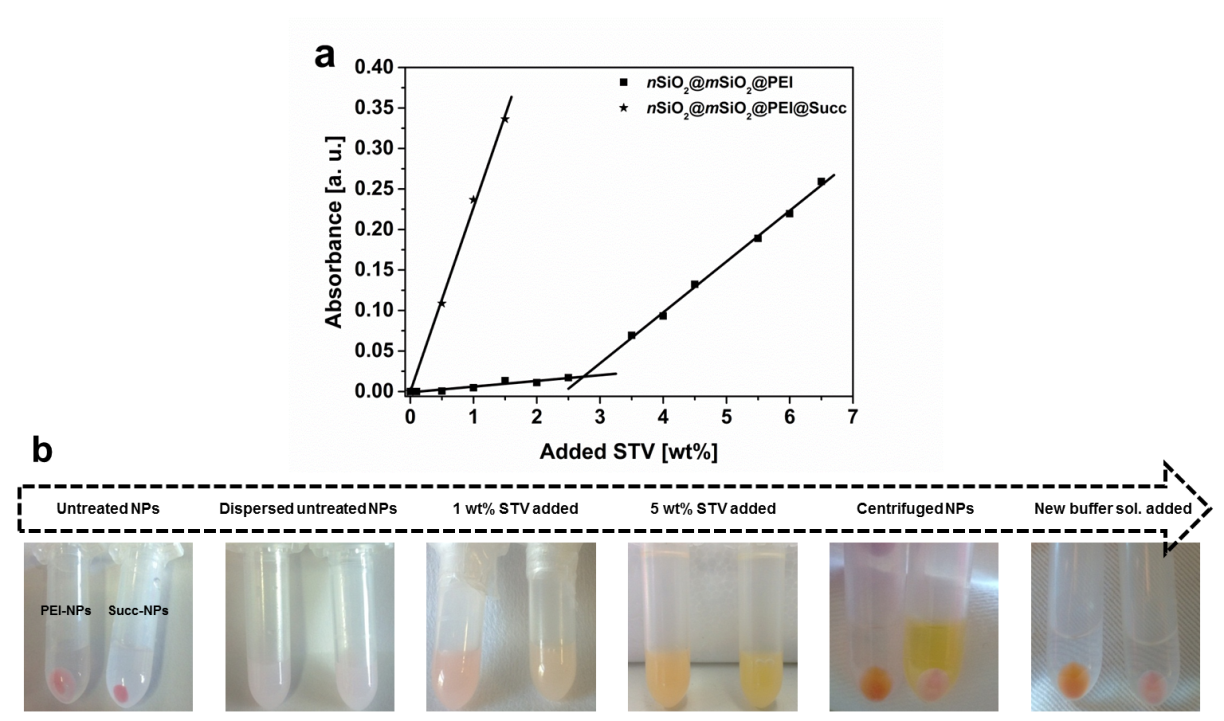


**Fig. S5** (a) Comparison of streptavidin (STV) adsorption to *n*SiO_2_@*m*SiO2@PEI particles (positively charged, +41 mV) and to *n*SiO_2_@*m*SiO_2_@PEI@Succ particles (negatively charged, -48 mV) in buffer solution at physiological pH (HEPES, pH 7.2), absorbance measured at 280 nm. Since streptavidin is negatively charged at physiological pH, no streptavidin was adsorbed on the *n*SiO_2_@*m*SiO_2_@PEI@Succ particles most likely due to electrostatic repulsion between the streptavidin and the particles. The images in (b) show the same result. Here, FITC-labeled streptavidin has been used and it can be clearly seen that a complete adsorption of 5 wt% streptavidin to *n*SiO_2_@*m*SiO_2_@PEI particles took place, while the yellow streptavidin was left in the supernatant for the *n*SiO_2_@*m*SiO_2_@PEI@Succ particles.


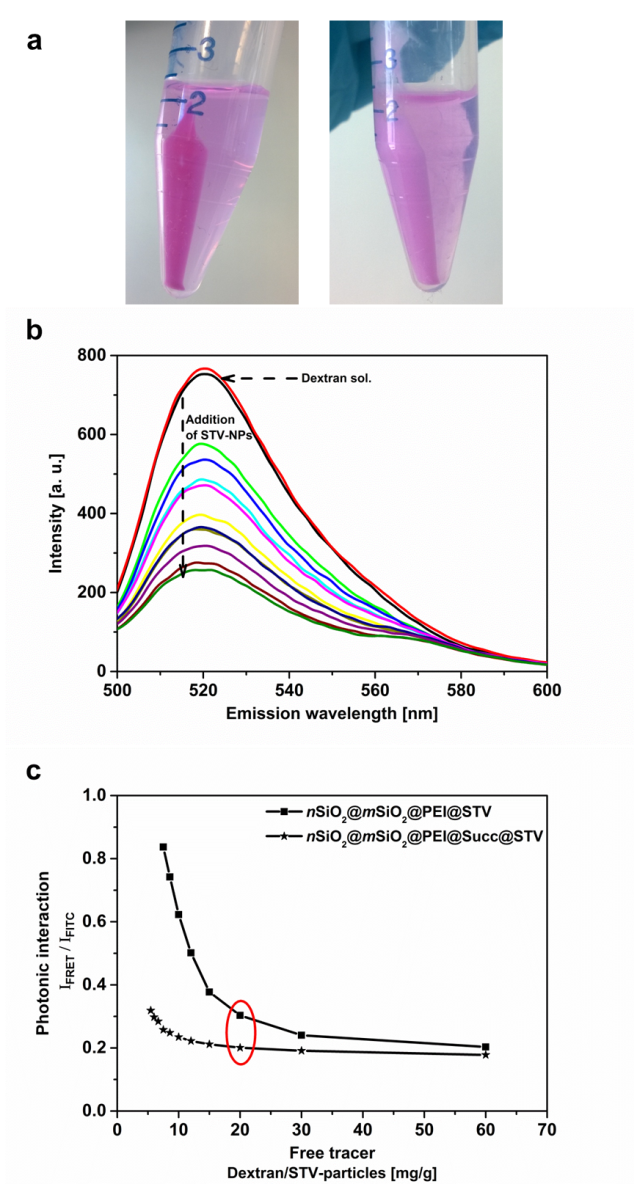


**Fig. S6** (a) Dylight 549-streptavidin labeled *n*SiO_2_@*m*SiO_2_@PEI particles (left) vs. *n*SiO_2_@*m*SiO_2_@PEI@Succ particles (right). Even if streptavidin did not adsorb on the negatively charged *n*SiO_2_@*m*SiO_2_@PEI@Succ particles, these particles could still be covalently conjugated with streptavidin, although not to the same degree as the *n*SiO_2_@*m*SiO_2_@PEI particles. (b) Fluorescence intensity measurements of the initial dextran solution, and after each addition of *n*SiO_2_@*m*SiO_2_@PEI@Succ@STV particles. (c) Comparison of the photonic interaction vs. free tracer of the *n*SiO_2_@*m*SiO_2_@PEI@Succ@STV particles and *n*SiO_2_@*m*SiO_2_@PEI@STV particles.
